# Aircraft engine sensor fault diagnostics using an on-line OBEM update method

**DOI:** 10.1371/journal.pone.0171037

**Published:** 2017-02-09

**Authors:** Xiaofeng Liu, Naiyu Xue, Ye Yuan

**Affiliations:** 1 School of Transportation Science and Engineering, Beijing University of Aeronautics and Astronautics, Beijing, PR China; 2 Collaborative Innovation Centre for Advanced Aero-Engine, Beijing, PR China; 3 Aircraft/Engine Integrated System Safety Beijing Key Laboratory, Beijing, PR China; 4 School of Energy and Power Engineering, Beijing University of Aeronautics and Astronautics, Beijing, PR China; Chongqing University, CHINA

## Abstract

This paper proposed a method to update the on-line health reference baseline of the On-Board Engine Model (OBEM) to maintain the effectiveness of an in-flight aircraft sensor Fault Detection and Isolation (FDI) system, in which a Hybrid Kalman Filter (HKF) was incorporated. Generated from a rapid in-flight engine degradation, a large health condition mismatch between the engine and the OBEM can corrupt the performance of the FDI. Therefore, it is necessary to update the OBEM online when a rapid degradation occurs, but the FDI system will lose estimation accuracy if the estimation and update are running simultaneously. To solve this problem, the health reference baseline for a nonlinear OBEM was updated using the proposed channel controller method. Simulations based on the turbojet engine Linear-Parameter Varying (LPV) model demonstrated the effectiveness of the proposed FDI system in the presence of substantial degradation, and the channel controller can ensure that the update process finishes without interference from a single sensor fault.

## Introduction

Because fault diagnostics are crucial for flight safety[1], several effective sensor FDI approaches have been developed recently[2, 3], such as fault detection observer in Takagi–Sugeno’s form, a bank of neural networks and so on. However, these approaches did not address how to maintain effectiveness when abrupt degradation occur. As engine output deviations increase due to the progression of health degradation, it becomes difficult to distinguish the presence of faults from the health degradation through an observation of the engine outputs. As a result, an inflight diagnostic system loses its effectiveness. Accurate online estimation is critically important, Zou *et al*. designed a multi-time-scale observer to realize accurate online state estimation for some types of nonlinear singularly perturbed systems[4]. To ensure accurate online estimation, the FDI system must be able to resist in-flight abrupt degradation and sensor fault.

A bank of Kalman filters was used to achieve engine sensor and actuator fault detection applied to a steady-state engine [5, 6]. However, it had limitations in an environment where various elements (such as the fuel input, health or flight condition) are changing (i.e., in the state transfer process). HKF is composed of a nonlinear on-board engine model (OBEM) and piecewise linear state-space models which include Kalman gain matrices. Because of this integration, the in-flight diagnostic system does not need to deal with engine health degradation and control input changes by itself. HKF possesses the numerical robustness of the piecewise linear Kalman filter approach and also the nonlinear estimation capability of the constant gain extended Kalman filter approach, therefore HKF can improve the estimation accuracy[7, 8]. The utilization of the nonlinear OBEM allows the reference health baseline of the in-flight diagnostic system to be updated to the degraded health condition of the engines through a relatively simple process. Without the health baseline update, any in-flight diagnostic system will lose its diagnostic effectiveness as the real engine degrades over its lifetime [9].

Kobayashi proposed that if the OBEM could track the engine health condition, the effectiveness of fault detection could be maintained. A method was developed to update the OBEM off-line [10]. The off-line OBEM-updating algorithm cannot ensure the accurate online state estimation when rapid degradation occurs.

The rapid degradation of an aeroengine can be caused by many factors. High-condensed sand or salt will accelerate the fan or the compressor degradation, while tropical heat will accelerate the turbine degradation [10]. The damage from foreign objects occurs primarily to the fan and other front parts of the aeroengine. The extent of the damage can be determined by the geometry, angle of impact, hardness, relative speed, etc. of the objects [11]. Volcanic ash might severely affect the hot sections of the aeroengine while producing no visible damage to the cooler portions in front of the engine, and ash ingestion could lead to a substantial component degradation[12]. When the OBEM was updated off-line, a mismatch between the OBEM and the actual engine still existed in flight because of the rapid degradation. Therefore, it is necessary to update the OBEM on-line.

To update the OBEM on-line, several approaches have been proposed recently [13–15]. Volponi presented an enhanced self-tuning on-board real-time model (eSTORM) to track the health deterioration of the aeroengine [16]. Simon et al. presented a real-time self-tuning model for engine performance monitoring and fault diagnosis [17, 18]. In those technologies, the following question remained: how to protect the OBEM updating procedure itself from a sensor fault.

To update the OBEM on-line, this paper proposes a new method that can be free from sensor fault and accommodate rapid degradation. However, the HKF will estimate incorrectly if the estimation and updating are running simultaneously (this will be explained in detail below). To solve this problem, a logic channel controller is designed. The proposed sensor FDI system is composed of a logic channel controller, a bank of HKFs, and a nonlinear OBEM.

In the following subsections of this paper, the design approach of an improved sensor FDI system based on updating the OBEM on-line is described. In this approach, a logic channel controller is designed and analysed in detail. Then, the design method is applied to an aircraft turbojet engine, and the performance of the proposed system is evaluated. The last part is the conclusion.

## Improved sensor fdi system based on updating the obem on-line

### Sensor fault detection system based on a hybrid Kalman filter structure

In this paper, an improved sensor FDI is established based on a nonlinear component-level engine model [19]. This nonlinear model is a single-spool turbojet engine model. The OBEM used in the FDI is a physics-based nonlinear model designed to run in real time. In order to compute Kalman gain matrices, the piecewise linear state-space models are derived off-line from the OBEM at the nominal health baseline. By a two-step perturbation linearization method [19], the nonlinear engine model can be linearized as:

$$\{\begin{array}{l} \;\Delta \dot{x}(t)=A\Delta x(t)+B\Delta u(t)+L\Delta h(t)+G\Delta z(t)\\ \Delta y_{m}(t)=C\Delta x(t)+D\Delta u(t)+M\Delta h(t)+H\Delta z(t) \end{array}$$ (1)

where ***x*** represents the variable states of the engine, ***y***_***m***_ represents the measured output parameters, and ***u*** is the control input of the engine. The augmented variable ***h*** represents the performance degradation factor, also called the health parameter. The vector ***z*** represents the measured parameters that define the flight condition, such as the altitude and Mach number. The matrices ***A***, ***B***, ***C***, ***D***, ***L***, ***M***, ***G*** and ***H*** are the corresponding state-space matrices that can be obtained from a partial derivative method [20, 21].

In order to estimate Δ***h*** via Δ***y***_***m***_, state variable model in Kalman filter should be augmented

$$\{\begin{array}{l} \Delta {\dot{x}}_{aug}=A_{aug}\Delta x_{aug}+B_{aug}\Delta u+v\\ \;\Delta y=C_{aug}\Delta x_{aug}+D_{aug}\Delta u+w \end{array}$$ (2)

where $\Delta x_{aug}=\left[\begin{array}{c} \Delta x\\ \Delta h \end{array}\right]$, $A_{aug}=\left[\begin{array}{cc} A & L\\ 0 & 0 \end{array}\right]$, $B_{aug}=\left[\begin{array}{c} B\\ 0 \end{array}\right]$, ***C***_***aug***_ = [***C M***], ***D***_***aug***_ = ***D***. ***w*** represents white process noise and ***v*** represents white measurement noise After optimal estimating by Kalman filter, Eq (2) can be obtained

$$\{\begin{array}{l} \Delta {\dot{\hat{x}}}_{aug}=A_{aug}\Delta {\hat{x}}_{aug}+B_{aug}\Delta u+K(\Delta y_{m}-\Delta {\hat{y}}_{m})\\ \;\Delta {\hat{y}}_{m}=C_{aug}\Delta {\hat{x}}_{aug}+D_{aug}\Delta u \end{array}$$ (3)

where ***K*** is Kalman gain matrix which obtained from Riccati equation[9].

This paper proposes an FDI combined with a group of HKFs. An HKF is composed of a nonlinear OBEM and piecewise linear state-space models which include Kalman gain matrices. The OBEM receives the estimated health condition off-line as a reference to minimize the deviation of the measured outputs between the OBEM and the actual engine.

The outputs of OBEM are:

$$\{\begin{array}{c} {\dot{x}}_{OBEM}=f(x_{OBEM},{\hat{h}}_{ref},u,z)\\ y_{OBEM}=g(x_{OBEM},{\hat{h}}_{ref},u,z) \end{array}$$ (4)

Vector ${\hat{h}}_{ref}$ represents the health condition reference baseline that feeds into the OBEM. It is traditionally calculated based on the post-flight data [7], which is an off-line method. This paper proposes an on-line method to update reference baseline ${\hat{h}}_{ref}$.

The HKF replaces the performance baseline of a linear Kalman filter with the outputs of the OBEM [7]. By integrating the OBEM and linear state-space matrices, the following HKF is formed:

$$\{\begin{array}{l} \Delta {\dot{\hat{x}}}_{aug}=A_{aug}\Delta {\hat{x}}_{aug}+K(y_{m}-{\hat{y}}_{m})\\ \;{\hat{y}}_{m}=C_{aug}\Delta {\hat{x}}_{aug}+y_{OBEM} \end{array}$$ (5)

where $\Delta {\hat{x}}_{aug}=\left[\begin{array}{c} \Delta \hat{x}\\ \Delta \hat{h} \end{array}\right]$, $\Delta \hat{x}=\hat{x}-x_{OBEM}$, $\Delta \hat{h}=\hat{h}-{\hat{h}}_{ref}$, ${\hat{y}}_{m}=\Delta {\hat{y}}_{m}+y_{OBEM}$. The control command inputs and associated matrices ***B***_***aug***_ and ***D***_***aug***_ in Eq (3) do not appear in Eq (5) since the effect of control command inputs is accounted for by the OBEM as seen in Eq (4).

The HKF in the LPV form[8] is:

$$\{\begin{array}{l} \left[\begin{array}{c} \Delta \hat{x}\\ \Delta \hat{h} \end{array}\right]=\left[\begin{array}{cc} A\left(\rho \right) & L\left(\rho \right)\\ 0 & 0 \end{array}\right]\left[\begin{array}{c} \hat{x}-x_{OBEM}\\ \hat{h}-{\hat{h}}_{ref} \end{array}\right]+K\left(\rho \right)(y_{m}-{\hat{y}}_{m})\\ {\hat{y}}_{m}=[\begin{array}{cc} C\left(\rho \right) & M\left(\rho \right) \end{array}]\left[\begin{array}{c} \hat{x}-x_{OBEM}\\ \hat{h}-{\hat{h}}_{ref} \end{array}\right]+y_{OBEM} \end{array}$$ (6)

where ***ρ*** is the parameter vector of the system that is not known in advance but can be measured or estimated in real-time, and where ***K***(***ρ***) is the Kalman filter gain

$$K(\rho )=P(\rho )C{\left(\rho \right)}^{T}R^{-1}$$ (7)

and ***P***(***ρ***) is the solution to the Riccati equation

$$A(\rho )P(\rho )+P(\rho )A^{T}(\rho )\text{-}P(\rho )C{\left(\rho \right)}^{T}R^{-1}C(\rho )P(\rho )+Q=0$$ (8)

The process noise diagonal matrix ***Q*** is related to the process noise column vector, and the measurement noise diagonal matrix ***R*** is related to the measurement noise vector [22].

Therefore, $\left(x_{OBEM},y_{OBEM},{\hat{h}}_{ref}\right)$ becomes the performance baseline of the HKF’s estimation, in which ***x***_*OBEM*_, ***y***_*OBEM*_, ${\hat{h}}_{ref}$ are the baseline state variables, baseline outputs, and the health baseline, respectively.

**Fig 1:**
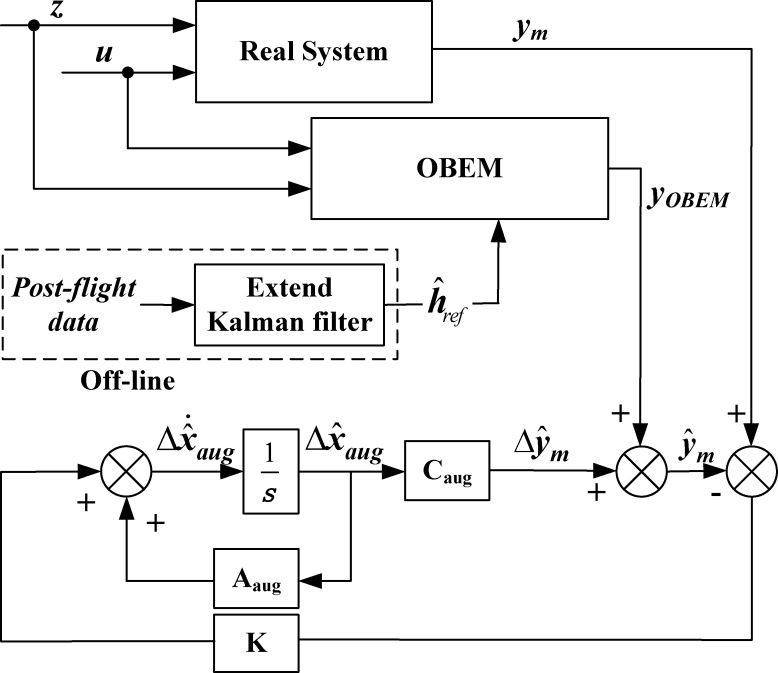
Structure of HKF.

An HKF does not need data training in advance to adapt to different situations, and its structure is simple [10], as shown in Fig 1. The OBEM is a baseline model, the measured outputs of the OBEM are used as the baseline measured outputs of the Kalman filter, and the engine performance factors can be estimated on-line by deviations in the measured outputs. The health baseline is updated through a relatively simple procedure by feeding the estimated health degradation values into the OBEM. Because of this hybrid structure, the in-flight diagnostic system does not need to deal with engine health degradation and control input changes by itself, therefore HKF can improve the estimation accuracy of the FDI system in the state transfer process.

**Fig 2:**
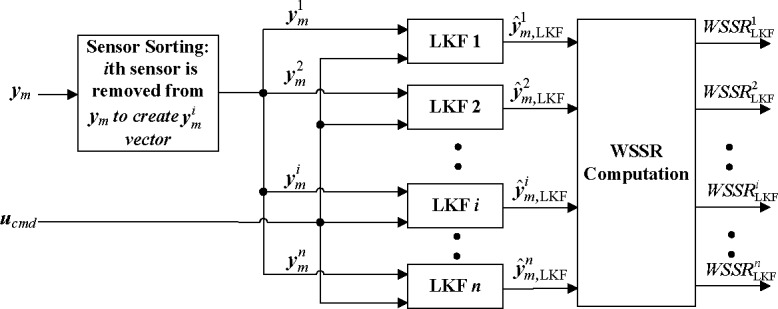
Structure of bank of LKFs.

**Fig 3:**
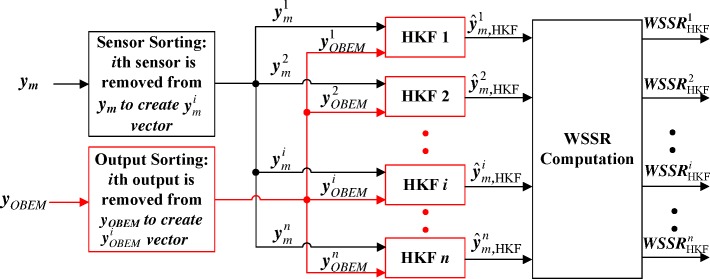
Structure of the bank of HKFs.

The conventional structure for the sensor FDI method using a bank of traditional linear Kalman filters(LKFs) is shown in Fig 2 [6]. To improve the estimation performance under dynamic conditions, an HKF is incorporated into the proposed structure, as shown in Fig 3. Two inputs to the bank of HKFs are the engine output signals (***y***_*m*_) and the OBEM output signals (***y***_*OBEM*_), while two inputs to the bank of LKFs are the engine output signals (***y***_*m*_) and the control commands (***u***). The effect of the control command inputs is accounted for by the OBEM.

Each HKF estimates the health parameter using *n-1* sensors and *n-1* OBEM outputs. In other words, the *i*th HKF uses the sensor subset $y_{m}^{i}$ that excludes the *i*th sensor, and the OBEM output subset $y_{OBEM}^{i}$ that excludes the *i*th OBEM output. The OBEM outputs are a one-to-one match to the sensor signals. The *i*th OBEM output subset $y_{OBEM}^{i}$ acts as the baseline outputs of the *i*th HKF.

The HKF performs better than the LKF, and these different performances result from their different structures. The LKF combines a piecewise linear engine model with a single Kalman gain matrix computed at a single operating point [23]. However, HKF incorporates a nonlinear OBEM to provide the baseline outputs, which can vary with different control commands and flight conditions, as shown in Eq (4) and Eq (5).

The differences between LKF and HKF are as followings. The control inputs and environmental inputs will bring parameter variations. In HKF, those variations are provided by OBEM while in LKF those variations are computed by filter itself. LKF needs to estimate the variation of outputs caused by health degradation, control inputs and environmental inputs, while HKF only needs to estimate the variation of outputs caused by health degradation.

The fault detection approach used for the bank of HKFs is composed of two steps. The first step is to compute the weighted sum of squared residual (WSSR) from the sensor measurements and the Kalman filter estimates as follows:

$$e_{i}={\hat{y}}_{m,\mathrm{HKF}}^{i}-y_{m}^{i}$$ (9)

$${WSSR}^{i}=W_{r}^{i}{\left(e^{i}\right)}^{\text{T}}{\left(\Sigma^{i}\right)}^{\text{-}1}e^{i}$$ (10)

$$\Sigma^{i}=diag{\left[\sigma^{i}\right]}^{2}$$ (11)

$$W_{r}{}^{i}={\left[y_{{}_{OBEM}}^{i}\right]}^{\text{-}2}$$ (12)

where ${\hat{y}}_{m,\mathrm{HKF}}^{i}$ is the sensor estimate generated by the *i*th HKF, *σ*^***i***^ represents the standard deviation of the *i*th sensor-subset, and *W*_*r*_^*i*^ is the weighting factor.

**Fig 4:**
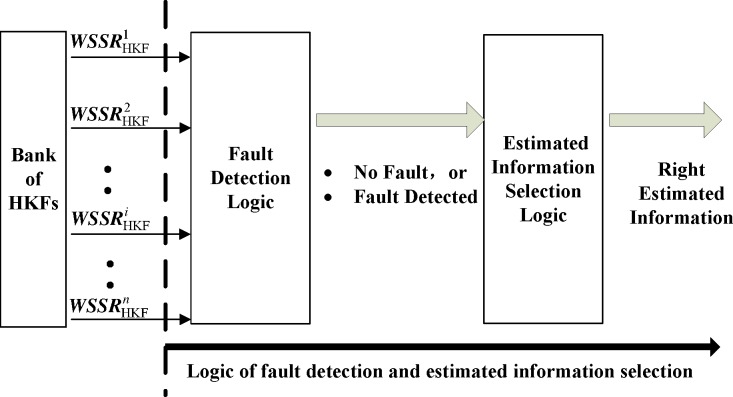
Logic of fault detection and estimated information selection.

The second step is to compare the residuals with the pre-established detection thresholds to detect a sensor fault. The logic of this fault detection and estimated information selection is designed as shown in Fig 4. It is assumed that only one of the sensors will fail at a time. The likelihood of multiple simultaneous failures is considerably low [5]. The threshold is discussed in Kobayashi’s paper [5].

The values of the *WSSRs* will move towards zero given the absence of a degradation and a fault [24]. When the *i*th sensor is faulty, although the *i*th HKF will be free from a faulty sensor signal, the rest of the filters will be influenced. Hajiyev *et al*. proposed that the statistics of a faulty sensor would have a greater deviation than those of the other sensors [25]. From the residual computational equations, the *i*th *WSSR* will be lower than the threshold, whereas the remaining *n*−1 residuals are higher than the threshold. Therefore, the faulty sensor can be located by Fault Detection Logic. Because the *i*th filter is able to estimate accurately, a set of accurate performance parameters can be obtained. If none of the *n* sensors are faulty, all filters can estimate accurately because they all use non-corrupted information. The Estimated Information Selection Logic can obtain estimated information accurately.

The bank of LKFs and the bank of HKFs are both able to estimate the variations of the health parameters for a single component degradation under the steady-state condition [5]. Because the HKF highly improves the estimation accuracy of the FDI system in the state transfer process [9], the HKF is incorporated in the proposed system. In combination with the selection logic, the bank of HKFs can provide accurate health information under the sensor fault or engine-state transfer conditions, which is the premise for an on-line update to the OBEM.

### Design of a logic channel controller for an on-line update to the OBEM

This paper assumes the absence of engine component faults or other faults of the engine itself, and therefore, only single sensor fault and rapid degradation are dealt with here. Incipient health degradation can be dealt with in the same method as rapid degradation.

#### Purposes

Updating the OBEM on-line is an effective approach to maintain the effectiveness of in-flight diagnostics for an aged engine [17]. However, there is a problem with this updating process. Updating the OBEM requires the HKFs’ estimated health information, which in turn is based on the outputs of the OBEM. The HKF estimation will lose its accuracy when the OBEM is updated simultaneously with the estimation. In other words, once the OBEM begins updating, the HKF should not use the OBEM’s outputs as a computation baseline until the OBEM is completely updated. Some actions must be taken to resolve this problem.

**Fig 5:**
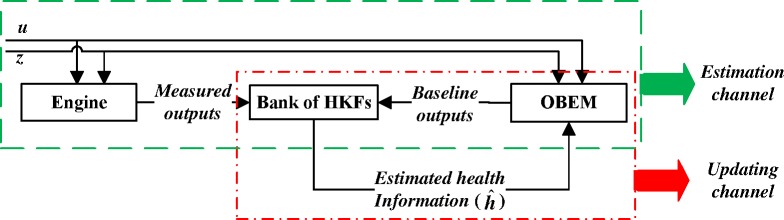
Two channels of processes which should not run simultaneously.

There are two process channels in the FDI system, as shown Fig 5. One is the estimation channel, which uses the deviation between the baseline outputs provided by the OBEM and the engine outputs to estimate the health information by a bank of HKFs. The other one is the updating channel, which filters the correct health information to update the health baseline of the OBEM, which OBEM then will generate new outputs.

The design goals of the logic channel controller are as follows:

1. Under incipient or slow health degradation conditions, the system runs in the estimation process channel. If the real engine suffers a rapid degradation, the channel will be switched to the updating channel, and then OBEM will reach the same degraded state as the real engine.
2. These two process channels are separated by the logic channel controller and will not disturb each other.
3. Regardless of whether there is a single sensor fault or not, the degradation can be estimated in real-time and the OBEM can be updated on-line.

#### Overall structure

**Fig 6:**
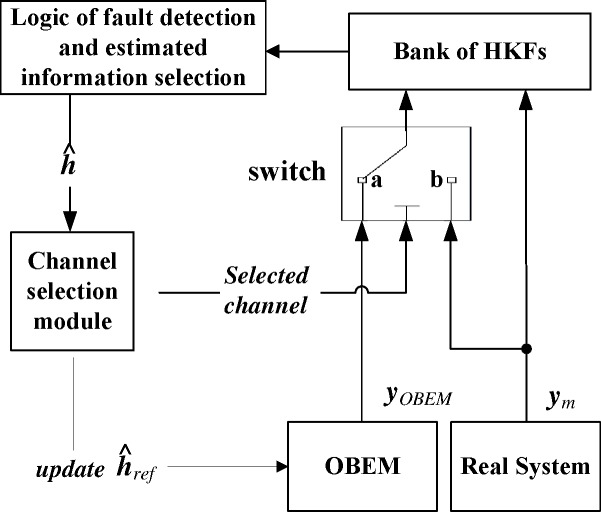
The overall structure of logic channel controller.

The overall structure of the logic channel controller is shown in Fig 6. The logic of fault detection and estimated information selection, called selection logic for short, is shown in Fig 5. In the switch, the following two alternative channels are provided: one is marked as “*a*” and represents the estimation channel; the other one is marked as “*b*”. When the channel selection module selects the “*b*” channel, ${\hat{h}}_{ref}$ will be updated at the same time, and then the system will proceed in the updating channel.

**Fig 7:**
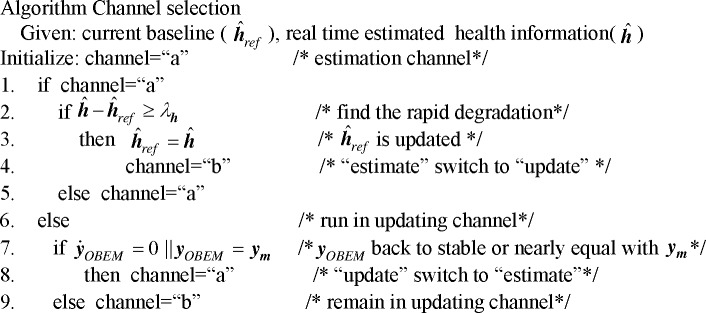
The algorithm of channel selection module.

The algorithm of the channel selection module is shown in Fig 7, where ***λ***_*h*_ is the threshold of the health parameter deviation. Kobayashi *et al*. divided the health condition mismatch into four levels, which range from 1% to 5% [7]. Because 5% is the maximum degree of degradation and 1~2% is relatively common in practical application, we chose 2.5% to be the threshold in this algorithm. The channel selection module has two types of switch commands.

(1) “Estimate to update” switch

The channel selection module has two input sets, the first of which is the difference between the estimated health information ($\hat{h}$) and the current health information baseline (${\hat{h}}_{ref}$). The degradation of the engine is unidirectional.

Because the degradation value is always growing with the flight time, the difference grows as well. Therefore, if $\hat{h}-{\hat{h}}_{\mathrm{ref}}\geq \lambda_{h}$, the “estimate” to “update” switch will be triggered. Otherwise, the health baseline of the OBEM will maintain the current value.

(2) “Update to estimate” switch

Whether to output an “update to estimate” command depends on the second input set, which are the outputs of the OBEM(***y***_*OBEM*_) and the engine outputs(***y***_*m*_). Depending on the engine states, two methods can be chosen for this decision-making.

Two types of engine states will be discussed. First, under steady states, the OBEM receives a new health baseline and its outputs gradually become stable. The estimation process will continue when the outputs of the OBEM become stable. Second, under transient states, the OBEM outputs should become nearly equal to the engine outputs, at which time the updating process is finished, and the channel can be switched back to the estimation process channel. The engine outputs here exclude the faulty sensor signal that is isolated by the FDI logic. While the second method for switch decision-making is also applicable to steady states, the first method is not applicable to transient states. However, the first method costs less effort than the second one in practical applications.

Note that updating the process channel does not mean simply feeding the real-time filters’ estimated health information into the OBEM. The right choice is to save the value of the health information provided by the selection logic at the moment the “*a*” to “*b*” switch occurs, then feed that value into the OBEM as a new health baseline. This new health baseline should maintain its value until the next “*a*” to “*b*” switch occurs.

#### Isolation of the two channels

When the updating process begins, the estimation of the Kalman filter will be inaccurate until the updating process finishes. Because the two channels have to be isolated, it would be perfect if the estimation could be “paused” when the updating channel is working. However, the Kalman filter does not have an inherent pause function. The switch in Fig 6 can solve this issue.

**Fig 8:**
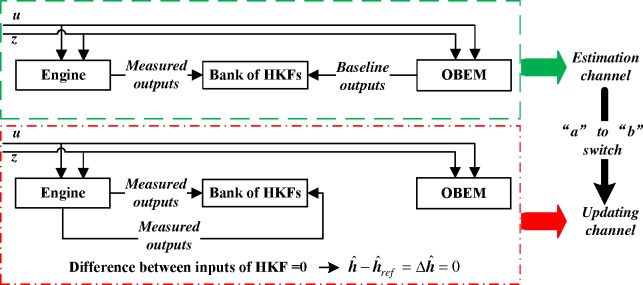
Two channels separated by the switch.

According to Eq (6), the computation of the Kalman filter is based on the difference between two inputs, namely the difference between ***y***_*OBEM*_ and ***y***_*m*_. After the switch, the difference is between ***y***_*m*_ and ***y***_*m*_, and therefore, the difference is changed to zero, and the estimation value of the augmented state variables $\Delta \hat{h}$ will also be changed to zero. As line No. 2 in Fig 7 shows, if $\hat{h}-{\hat{h}}_{ref}$ exceeds the threshold, the switch will be triggered. Once the switch is triggered, the health baseline would be replaced by the new estimated value. However, the estimation will be incorrect if the update and the estimation are run simultaneously. In the proposed system, the mathematical expressions in Fig 8 show that when the updating process begins, $\hat{h}-{\hat{h}}_{ref}$ will become zero. Because this zero value will never exceed threshold when the update begins, the switch will not be triggered, and then the current health information baseline will not be replaced by the incorrectly estimated health information. This is the purpose of the “pause” function. When the update begins, the values of the estimated state variables are zeros, then the estimation process can be approximately regarded as being “paused” by the switch.

When switched back to a new estimation process channel, the bank of HKFs will receive the two original inputs again to continue an accurate estimation process. With the two separated channels, the OBEM update can finish without the corruption of the estimation process, and each estimation process can more accurately estimate based on the updated baseline outputs.

When the update is complete, the estimated health condition becomes the new reference baseline for the in-flight diagnostic system. The update time is very short, around a few tenths of a second. Compared with the time of the estimation and updating processes, the cruise time is extremely longer. The updating process costs even less time than the estimation. This in-flight channel-switching cycle can be periodically repeated, and then the OBEM can be updated on-line.

The OBEM proposed in this paper is different from the on-board model in previous studies due to the following two main differences: (1) the nonlinear on-board model is updated with this new channel controller method and (2) the channel controller can protect the update process of the OBEM from sensor fault.

## Simulation results

The performance of the proposed sensor FDI system based on an on-line OBEM update is evaluated by applying it to the engine model simulation.

**Fig 9:**
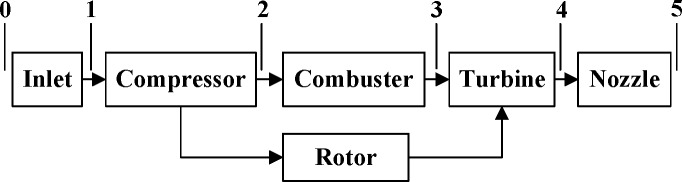
Schematic configuration of single-spool turbojet engine.

The engine model used in this paper is a nonlinear simulation of a single-spool turbojet engine that is in an LPV structure [26]. This engine model has been constructed as a Component Level Model (CLM), which consists of the major components of an aircraft engine. The CLM represents the highly complex engine physics and is designed to run in real time. The schematic configuration of this turbojet engine is shown in Fig 9, which includes the aeroengine compressor (*AC*), combuster, aeroengine turbine (*AT*), and the other components. The *AC* and the *AT* are on the same shaft driven by the rotor. Meanwhile, the degradation of engine performance is modelled using adjustments to the efficiency or to the flow coefficient scalars of the *AT* and the *AC*. These scalars representing the component performance deterioration are the health parameters.

The nonlinear engine model is used to represent both the actual engine and the OBEM in the subsequent sections [9]. The engine model representing the actual engine operates under the given health conditions, and its flight condition is specified by the three environmental parameters listed in Table 1. Symbols of the single-spool turbojet engine are listed in Table 2. The OBEM operates under the estimated health conditions that are fed in by the designed logic channel controller, and its flight conditions are specified by the following three measured parameters: ***T***_***amb***_, ***P***_***amb***_, and ***T***_***inlet***_. From these three measurements, the OBEM calculates the altitude and Mach number from the standard day condition. The actual engine and the OBEM receive the same control commands.

**Table 1:** Engine model variables.

State variables	*N*
Sensors	*N*, *P*_*2*_, *T*_*2*_, *P*_*4*_ and *T*_*4*_
Health parameters	*AC* efficiency, *AC* flow capacity, *AT* efficiency, *AT* flow capacity
Actuator	Fuel flow
Environmental Parameters	Altitude, Mach number, Ambient temperature

**Table 2:** Symbols of the single-spool turbojet engine.

Symbols and meaning	
*N*	rotor speed
*P*_*2*_	compressor exit pressure
*P*_*4*_	turbine exit pressure
*P*_*amb*_	Ambient pressure
*T*_*2*_	compressor exit temperature
*T*_*4*_	turbine exit temperature
*T*_*amb*_	Ambient temperature
*T*_*inlet*_	engine inlet temperature

To show the variations of health parameters directly, the degradation coefficient (*DC*) of the health parameters is used to represent the ratio of the degraded health parameters to the nominal health parameters. For example, the *DC* of *E*_*AT*_ is shown in the following equation, where *E* means effectiveness and *F* means flow capacity:

$$DC_{E_{AT}}=E_{AT,\mathrm{degradation}}/E_{AT,\text{nominal}}$$ (13)

The augmented state variables of the HKFs are $x_{aug}={\left[\begin{array}{ccccc} N & DC_{E_{AC}} & DC_{F_{AC}} & DC_{E_{AT}} & DC_{F_{AT}} \end{array}\right]}^{T}$.

One steady state point of the aeroengine is *x*=9545.0344. The matrices are

$$A_{aug}=\left[\begin{array}{ccccc} \text{-}21.1653 & \text{-}1.2796\times 10^{5} & 3373.7314 & \text{-}1.4379\times 10^{5} & 1.7702\times 10^{5}\\ 0 & 0 & 0 & 0 & 0\\ 0 & 0 & 0 & 0 & 0\\ 0 & 0 & 0 & 0 & 0\\ 0 & 0 & 0 & 0 & 0 \end{array}\right],$$

$$C_{aug}=\left[\begin{array}{ccccc} 1 & 0 & 0 & 0 & 0\\ 25.3890 & 2375.0619 & \text{-}41329.9975 & 304.7110 & 59877.1873\\ 1.62\times 10^{-2} & 85.1299 & \text{-}0.7910 & 6.91\times 10^{-2} & 0.9505\\ 4.6977 & 539.7504 & \text{-}7796.3228 & 1800.6119 & \text{-}24654.1862\\ \text{-}0.1345 & 108.4028 & 252.7247 & 108.4922 & 607.8732 \end{array}\right].$$

The values of ***Q*** and ***R*** are set to ***Q*** = 1***e***−5***I***_5×5_, ***R*** = 1***e***−5***I***_5×5_, and the gain matrix ***K*** is computed from the above matrices. The values of ***Q*** and ***R*** are discussed in reference [8].

$$K=\left[\begin{array}{ccccc} 881.8709 & 9.4691 & \text{-}206.4104 & \text{-}16.0700 & \text{-}753.1645\\ -2.5862 & 1.0425 & 28.0233 & 1.5390 & 14.3022\\ 0.1614 & -22.0515 & 0.4475 & -22.4373 & 3.1739\\ -2.8263 & 0.3575 & -14.5982 & 3.2577 & 27.7157\\ -0.1454 & 22.6363 & -0.6387 & -21.9864 & 1.9410 \end{array}\right]$$

Another steady-state point is ***x*** = 10899.1825, and the matrices are

$$A_{aug}=\left[\begin{array}{ccccc} \text{-}27.3637 & \text{-}1.6636\times 10^{5} & 10749.4758 & \text{-}1.9200\times 10^{5} & 2.4374\times 10^{5}\\ 0 & 0 & 0 & 0 & 0\\ 0 & 0 & 0 & 0 & 0\\ 0 & 0 & 0 & 0 & 0\\ 0 & 0 & 0 & 0 & 0 \end{array}\right],$$

$$C_{aug}=\left[\begin{array}{ccccc} 1 & 0 & 0 & 0 & 0\\ 31.3143 & 3723.0690 & -58091.4895 & 150.8606 & 84875.8452\\ 1.79\times 10^{-2} & 108.5529 & -0.2007 & 0.1227 & 0.1587\\ 6.4111 & 947.7220 & -12163.7968 & 3244.5356 & -36750.4900\\ \text{-}0.1296 & 134.7593 & 282.5733 & 137.3763 & 619.3872 \end{array}\right].$$

The values of ***Q***' and ***R***' are set to ***Q***' = 1***e***−5***I***_5×5_, ***R***' = 1***e***−5***I***_5×5_, and the gain matrix ***K*** is computed from the above matrices.

$$K=\left[\begin{array}{ccccc} 916.2049 & 9.1036 & \text{-}218.3181 & \text{-}10.8496 & \text{-}797.7607\\ -2.1603 & 1.2187 & 28.1373 & 1.6213 & 14.1248\\ 0.1287 & -22.0653 & 0.5316 & -22.3837 & 3.4339\\ -2.4085 & 0.3876 & -14.3787 & 3.5329 & 27.8356\\ -0.1508 & 22.6139 & -0.7627 & -21.9937 & 2.0696 \end{array}\right]$$

A Kalman filter with LPV structure in the HKF can be constructed based on the family of linearization data and functions between the elements of the matrices of the Kalman filter and the scheduling parameter. The following simulations show how the method is applied to the aeroengine.

In this example, ***ρ*** = *N* is chosen to be the scheduling parameter in the LPV model, and there would be functions between the family of *N* and the family of the elements of the matrices at different steady-state points.

To prove that HKF can improve the estimation accuracy of the FDI system in state transfer process, the following simulation is performed.

The simulation uses same model as the revised manuscript. It is conducted in transient operating conditions. At *t*=0.5s, the fuel input would increase by 14%, which would cause aeroengine acceleration. Then at *t*=1s, $DC_{E_{AC}}$ would decrease by 1%.

**Fig 10:**
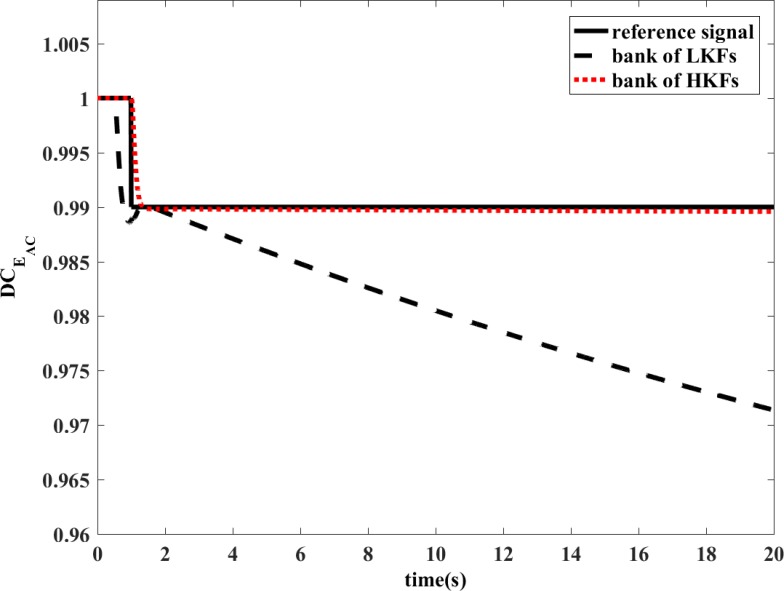
Comparison of the estimated value of $DC_{E_{AC}}$.

**Fig 11:**
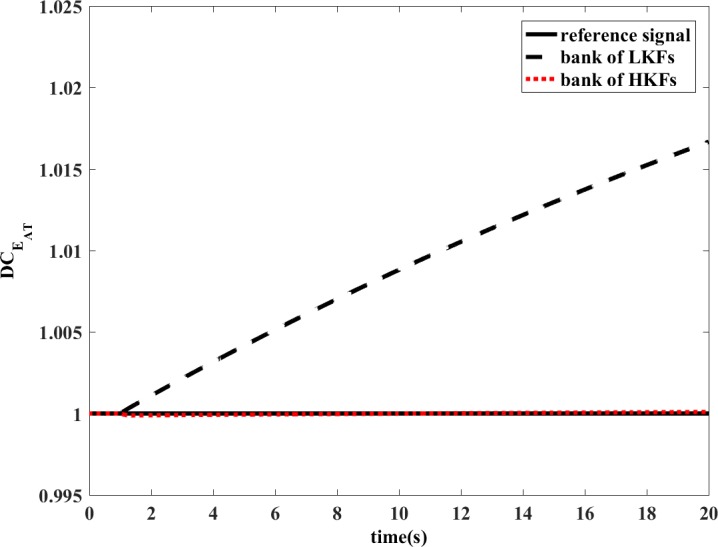
Comparison of the estimated value of $DC_{E_{AT}}$.

**Fig 12:**
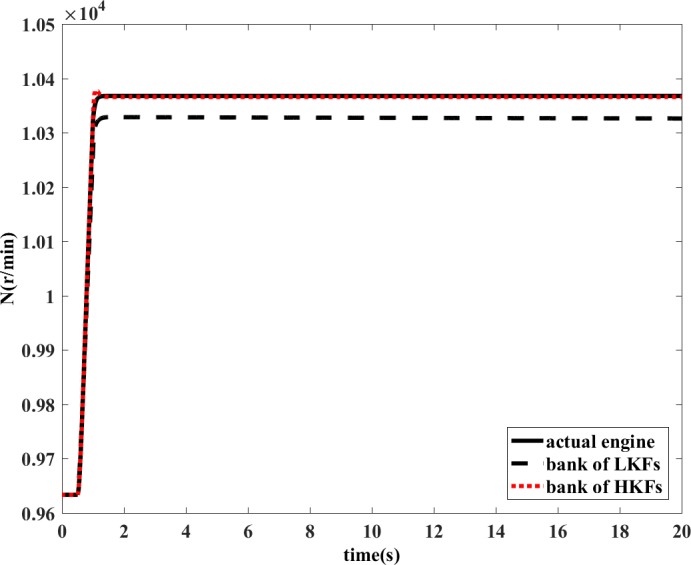
Comparison of the estimated value of *N*.

**Fig 13:**
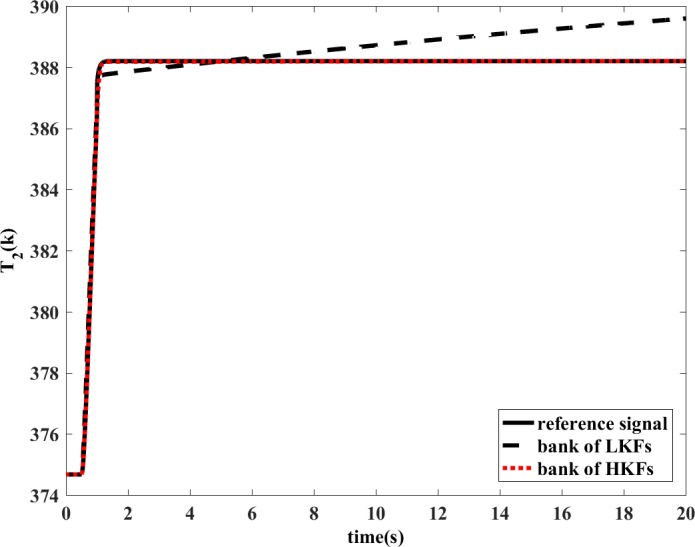
Comparison of the estimated value of *T*_2_.

The bank of LKFs and the bank of HKFs are both able to estimate the variation of health parameters in the situation of single component degradation in steady-state condition. However, the design of LKF is not applicable to transient operating conditions. The degradation coefficient of health parameter is shown in Fig 10 and Fig 11. In the dynamic process, the estimation accuracy of LKF would be even wrong, while the HKF would still remain its estimation accuracy. The estimations of measured outputs are shown in Fig 12 and Fig 13. Obviously, the deviations of *N* and *T*_2_ between LKF and engine are larger than those between HKF and engine.

The performance of the logic channel controller is evaluated under the steady-state condition, where the engine is suffering a single substantial component degradation. At *t* = 1s, $DC_{E_{AT}}$ would decrease by 3%, and one of the sensors is failed at the same time. The variations of the health parameters are a step input.

**Fig 14:**
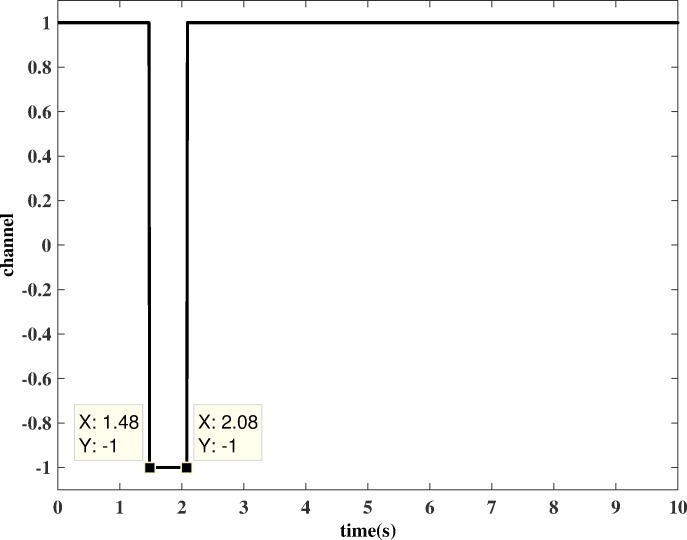
Channel switch signal (“1”: estimation channel; “-1”: updating channel).

The complete channel-switching process is finished in the simulation time as shown in Fig 14. Here “1” represents the estimation channel and “-1” represents the updating channel. At first, the system is running in a normal estimation process. When the switch is set to the “*a*” port, the degradation of the engine is estimated by a bank of HKFs, and the residuals of each HKF are computed. Suddenly, one of the engine sensors is failed. By analysing the residuals, the selection logic can locate the faulty sensor, select the correctly estimated health information, and feed this information to the channel selection module. At *t*=1.48 s, the difference between this health information and the current health baseline of the OBEM is larger than the threshold, and therefore, the channel selection module detects a substantial component degradation, and the “estimate to update” switch is triggered. Then, the health baseline of the OBEM is updated by the health parameters estimated at the moment the switch occurs. Meanwhile, the switch is now set to the “*b*” port, and the difference between the inputs of the bank of HKFs is changed to zero, which means that the estimation process can be regarded as paused and will not continue until the update process is completed. The “update to estimate” switch of the channel selection module is triggered at *t*=2.08 s, which indicates that the update is finished. The OBEM reaches the same state as the actual engine, and the switch in Fig 7 is set back to the “*a*” port. The outputs of the updated OBEM and the sensor signals of the engine are fed into the bank of HKFs to continue the estimation. The system goes back to the estimation channel, and therefore, the OBEM has been successfully updated on-line.

**Fig 15:**
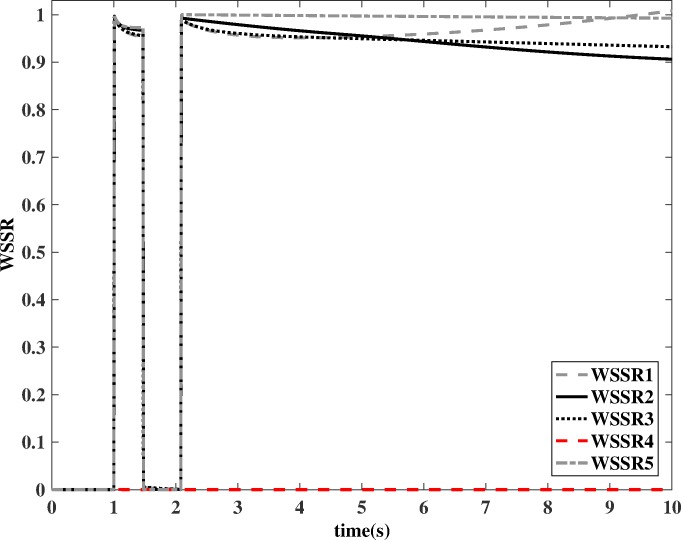
Values of *WSSR* with a fault in *P*_4_ sensor and $DC_{E_{AC}}$ decreased by 3%.

As mentioned earlier, the sensor signal that is not used by a particular filter is the one being monitored by that filter. *WSSR1*, *WSSR2*, *WSSR3*, *WSSR4* and *WSSR5* are the residuals generated by HKFs that monitor sensors *N*, *P*_*1*_, *T*_*2*_, *P*_*4*_ and *T*_*4*_, respectively. As shown in Fig 15, *WSSR1*, *WSSR2*, *WSSR3* and *WSSR5* grow rapidly whereas *WSSR4* remains nearly unchanged, which indicates a fault in the aeroengine turbine outlet pressure sensor. As for the sudden fall of all the *WSSR*s during the update, the reason is that while updating the channel, the differences between the two set of inputs of the HKF are set to zero, and therefore, zero is the value of the residuals. The fault detection procedure is already completed in the first estimation process, and therefore, it will not be affected.

**Fig 16:**
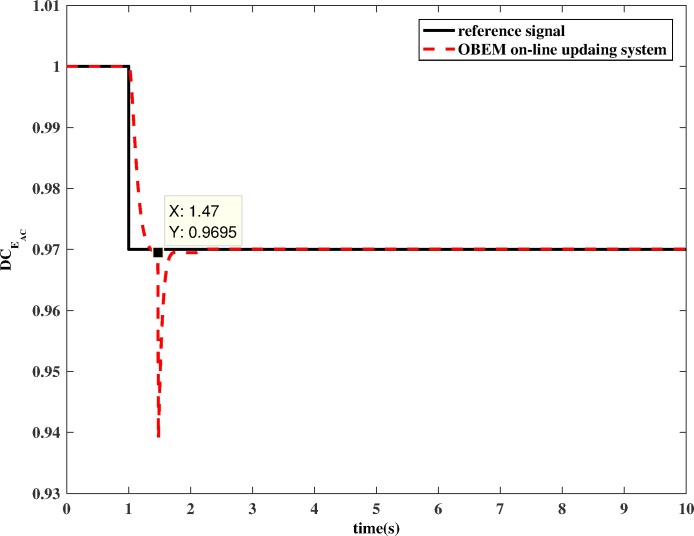
Estimation performance of OBEM on-line updating method (example of $DC_{E_{AC}}$).

**Fig 17:**
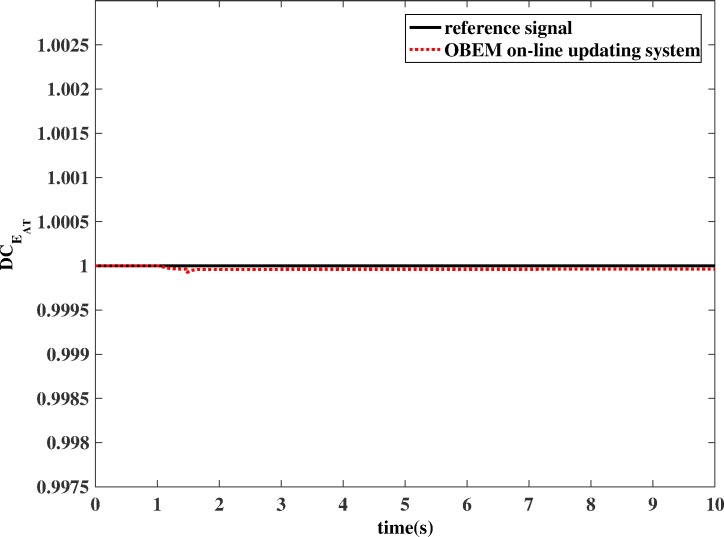
Estimation performance of OBEM on-line updating method (example of $DC_{E_{AT}}$).

In addition to locating the faulty sensor, the system can estimate the health parameters accurately. After identifying the faulty sensor, the Kalman filter can generate estimated information accurately, which is then selected and fed to the OBEM. The estimated health parameters are shown in Fig 16 and Fig 17. There exists a sudden fall in the $DC_{E_{AC}}$ when the update process begins for reasons that are presented below. To ensure the switch is not incorrectly triggered, the logic controller sets the deviation ($y_{m}-{\hat{y}}_{m}$ in Eq (6)) to be zero during the update process. However, the last instantaneous estimated $\Delta {\hat{x}}_{aug}$ is non-zero at the moment the channel switches, and therefore, $y_{m}-{\hat{y}}_{m}$ is non-zero, which is why the sudden fall exists. Once the switch is triggered, the two inputs of the HKF will be same because the initial input ***y***_*OBEM*_ will be replaced by ***y***_***m***_. In other words, the HKF will regard the OBEM to be exactly the same as the actual engine with no deviation between them. The characteristic of the Kalman filter is to fit the estimation value to the actual value, and the estimated deviation $\Delta {\hat{x}}_{aug}$ will return to zero quickly, which is why the fall only occurs for a very short time. This fall does matter because once the “estimate to update” switch is triggered; the new estimated health information will be saved by the channel selection module. The health baseline of the OBEM will maintain this new value during the update, and it will not be influenced by the following estimation values until the update is completed.

**Fig 18:**
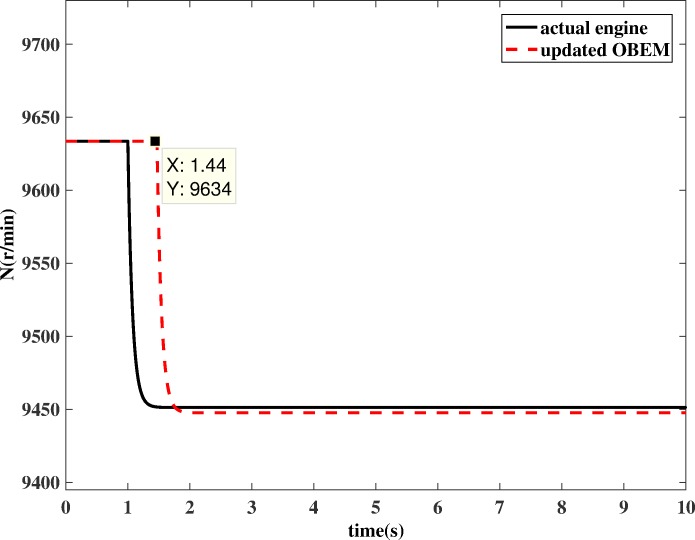
*N* comparison between actual engine and updated OBEM.

**Fig 19:**
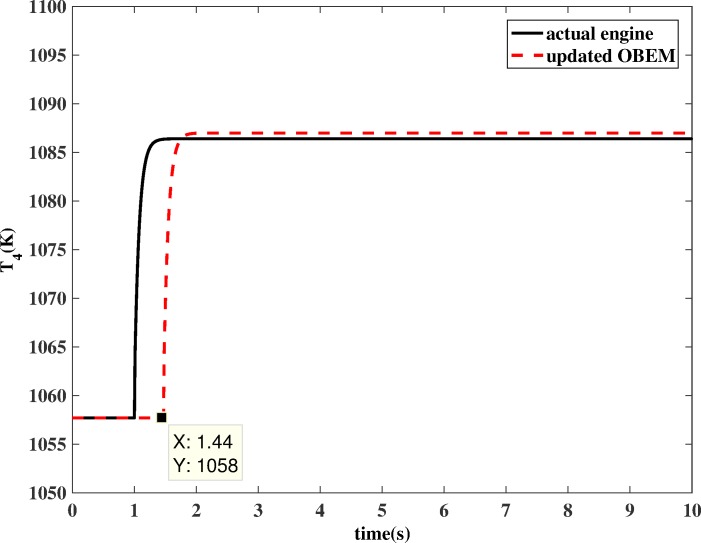
*T*_4_ comparison between actual engine and updated OBEM.

If OBEM is not updated, the outputs of the OBEM will maintain their off-line set values, which means the OBEM will not able to react to actual in-flight degradation. As shown in Fig 18 and Fig 19, the simulation result shows that the updated OBEM tracks the state of the actual engine. Therefore, the channel controller can enable the OBEM to track the engine’s health condition on-line. The steady`-state error of *N* is 0.03%, and the steady-state error of *T*_*4*_ is 0.09%. The short delay is the time cost of the update, only a few tenths of a second, which is significantly shorter than the engine running time, which is usually several hours for one flight. The delay time can also be compared to the adjusting time of the Kalman filter. The adjusting time of the Kalman filter is from the component degradation appears to it is detected by Kalman filter. Fig 16 shows the adjusting time of the Kalman filter is 0.47 s, and Fig 18 shows the delay time is 0.44 s. The Kalman filter is a real-time estimator, and the delay is short. The updating process costs even less time than the estimation. The sensor fault detection, which is based on the estimation channel, will not be influenced because the estimation process and updating process are separated by the channel controller.

## Conclusions

1. The designed improved sensor fault detection and isolation system uses an on-line method to update the health reference baseline of the on-board engine model. In this system, a Hybrid Kalman Filter is incorporated to improve accuracy in the state transfer process, and a logic channel controller is designed.
2. The results prove that the on-line update to the on-board engine model is protected by the estimated information selection logic and the logic channel controller. If a sensor fault occurs, the estimated information selection logic will approximate the correct information, isolating the faulty signals. This accurate information is used by the channel controller to update the reference baseline of the on-board engine model. Then, the on-board engine model can generate new proper outputs to act as the estimation baseline of the HKF. The updating and estimation processes are separated by a logic channel controller to protect the updating process from a potential false estimation of the HKF.
3. Evaluations conducted under rapid degradation conditions demonstrate that the on-board engine model can be updated on-line successfully to track the real engine health conditions, which can maintain the effectiveness of the fault detection.
